# Prevalence of Dental Caries in Children of Age 5 to 13 Years in District of Vaishali, Bihar, India

**DOI:** 10.5005/jp-journals-10005-1540

**Published:** 2018-10-01

**Authors:** Puneet Goenka, Samir Dutta, Nikhil Marwah, Aditi Sarawgi, Mitakshara Nirwan, Pooja Mishra

**Affiliations:** 1 Associate Professor, Department of Pediatric and Preventive Dentistry, Mahatma Gandhi Dental College & Hospital, Jaipur, Rajasthan, India; 2 Senior Professor, Department of Pedodontics, Government Dental College, Rajasthan, India; 3 Professor, Department of Pedodontics, Mahatma Gandhi Dental College & Hospital, Jaipur, Rajasthan, India; 4 Senior Lecturer, Department of Prosthodontics, Mahatma Gandhi Dental College & Hospital, Jaipur, Rajasthan, India; 5 Postgraduate Student, Department of Pedodontics, Mahatma Gandhi Dental College & Hospital, Jaipur, Rajasthan, India; 6 Postgraduate Student, Department of Pedodontics, Mahatma Gandhi Dental College & Hospital, Jaipur, Rajasthan, India

**Keywords:** Children, Dental caries, Prevalence, Vaishali.

## Abstract

**Context:**

Dental caries is the most common type of oral health problem globally. It is known to have multifactorial etiology with a number of variables that influence the prevalence of the condition.

**Aim:**

The present study was carried out in the district of Vaishali, Bihar, India, with an aim to determine the prevalence of dental caries in children of 5 to 13 years.

**Settings and design:**

It was a descriptive type of epidemiological study and the design adopted for the study was cross-sectional. No active intervention and follow-up examinations were performed.

**Materials and methods:**

A total of 1,000 children of 5 to 13 year age group were examined for the study. The study population was categorized based on age, sex, location, and socioeconomic status. The examination procedure and criteria were those recommended by the World Health Organization (WHO).

**Statistical analysis used:**

The data obtained from the survey were subjected to statistical evaluation using the Statistical Package for the Social Sciences (SPSS) software. Test for significance was done with the help of analysis of variance (ANOVA) and chi-square test.

**Results:**

The difference in the caries prevalence between the age groups and between the socioeconomic level was very highly significant (p = 0.000). There was a statistically significant difference observed in the prevalence of caries between the sexes (p = 0.016) as well as between urban and rural (p = 0.018).

**Conclusion:**

It is expected that the data obtained with the help of this survey will prove to be very useful to the concerned authorities in handling dental caries which is a biosocial disease rooted in the technology and economy of our society.

**How to cite this article:** Goenka P, Dutta S, Marwah N, Sarawgi A, Nirwan M, Mishra P. Prevalence of Dental Caries in Children of Age 5 to 13 Years in District of Vaishali, Bihar, India. Int J Clin Pediatr Dent 2018;11(5):359-364.

## INTRODUCTION

Dental caries is the most common type of oral health problem globally. Despite credible scientific advances and the fact that caries is preventable, the disease continues to be a major public health problem. In developing countries, changing lifestyles and dietary patterns are markedly increasing incidence of caries.^1,2^

Dental caries is known to have multifactorial etiology with a number of variables that influence the prevalence of the condition. In the past, innumerable studies and surveys have been conducted to determine the prevalence of the disease and the variables associated with its prevalence across the globe. Still a number of towns and districts lack data on the prevalence of oral health problems which is very essential to formulate an action plan to combat them. Vaishali is one such district which is located in the center of north Indian state of Bihar. This prompted us to conduct a study with limited resources in various government and nonaided public schools of the district so as to chart out the magnitude of the problem among pediatric population in the district. It is expected that the data obtained with the help of this survey will prove to be very useful to the concerned authorities in handling this biosocial disease rooted in the technology and economy of our society.

## MATERIALS AND METHODS

The present study was carried out in the district of Vaish-ali, Bihar, India, with an aim to determine the prevalence of dental caries in children of 5 to 13 years. A total of 1,000 children of 5- to 13-year age group, attending various schools of the district were examined for the study.

The study population was divided into high and low socioeconomic status based on the type of school attended. Children attending government schools were considered to belong to low socioeconomic status, whereas those attending nonaided schools were considered to belong to high socioeconomic status.

**Table 1:** **Table 1:** Prevalence of dental caries according to age

				*Children free of caries*				*Children with caries*				*dmft*				*DMFT*			
*Age group*		*No. of children examined*		*N*		*%*		*N*		*%*		*Mean*		*SD*		*Mean*		*SD*	
5-7		312		109		34.9		203		65.1		2.68		2.48		0.75		1.02	
8-10		353		153		43.3		200		56.7		2.01		2.07		1.05		1.28	
11-13		335		183		54.6		152		45.4		0.69		1.07		1.26		1.50	

SD: Standard deviation; Caries prevalence: Chi-square test = 25.657, p = 0.000; dmft: ANOVA, F = 86.970, p = 0.000; DMFT: ANOVA, F = 12.954, p = 0.000

It was a descriptive type of epidemiological study and the design adopted for the study was cross-sectional, i.e., the study subjects were examined only once in the study period to determine the prevalence and severity of the disease. No active intervention and follow-up examinations were performed.

### Procedure

Consent for examining the children was obtained from the respective parents. The children were examined on an upright chair in adequate natural light. Examination of the child was done by single trained and calibrated examiner to avoid any interexaminer variability. Data were recorded by a trained assistant. The examination procedure and criteria were those recommended by the WHO.^3^

### Data Entry and Statistical Evaluation

Decayed, missing, and filled teeth (DMFT) and decayed, extracted, and filled teeth (deft) values were calculated from the WHO forms filled after the examination of each patient and were entered in Microsoft Office Excel 2007 worksheet. The data obtained from the survey were subjected to statistical evaluation using the SPSS software.

## OBSERVATIONS AND RESULTS

### Prevalence of Dental Caries According to Age Groups

A total of 1,000 school-going children of 5- to 13-year age group were examined to find out the prevalence of dental caries among them. The study population was divided into three groups with 312 (31.2%) children belonging to 5 to 7 years age, 353 (35.3%) belonging to 8 to 10 years age, and 335 (33.5%) children belonging to 11- to 13-year age group.

In the 5- to 7-year age group, 109 (34.9%) children were found to be caries-free, while 203 (65.1%) showed one or more carious lesions. The mean dmft of the group was 2.68 ± 2.48, whereas the mean DMFT was recorded as 0.75 ± 1.02. In the 8- to 10-year age group, the prevalence of dental caries was found to be 56.7%. The dmft and DMFT scores were 2.01 ± 2.07 and 1.05 ± 1.28 respectively; 45.4% of the children in the 11- to 13-year age group had caries involved teeth. The mean dmft was 0.69 ± 1.07, whereas the mean DMFT score was 1.26 ± 1.50.

**Fig. 1:**
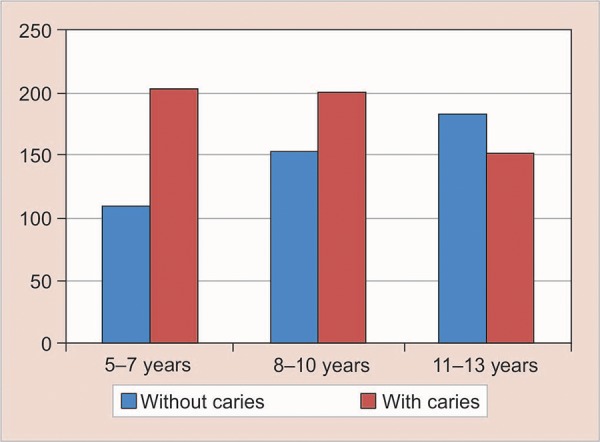
Prevalence of caries according to age

The difference in the caries prevalence between the age groups was very highly significant (p = 0.000). The difference in the dmft/DMFT scores among the three age groups was also statistically highly significant (p = 0.000). The dmft scores declined progressively as the age advanced, whereas the DMFT scores increased from 5 to 13 years (Table 1 and Fig. 1).

### Prevalence of Dental Caries According to Sex

**Fig. 2:**
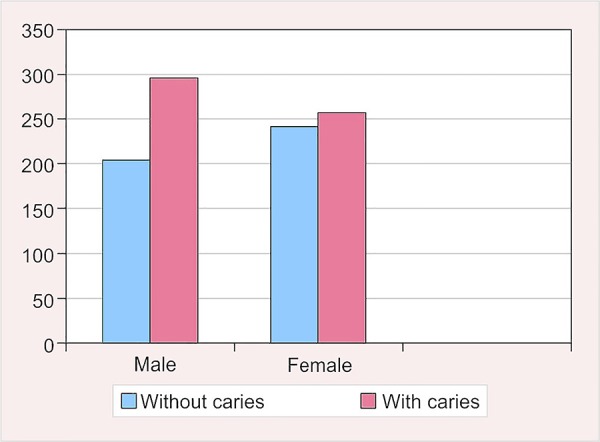
Prevalence of caries according to sex

Out of 1,000 children examined, 501 (50.1%) were males and 499 (49.9%) were females. The caries prevalence among males was found to be 59.3% with the mean dmft and DMFT score of 1.92 ± 2.09 and 1.21 ± 1.35 respectively. At the same time, 51.7% of females were found to be affected by dental caries with a mean dmft and DMFT scores of 1.63 ± 2.13 and 0.85 ± 1.23 respectively (Table 2 and Fig. 2).

There was a statistically significant difference observed in the prevalence of caries (p = 0.016) between the sexes. The difference in the dmft (p = 0.030) and DMFT scores was also found to be statistically significant.

### Prevalence of Dental Caries According to Location

**Fig. 3:**
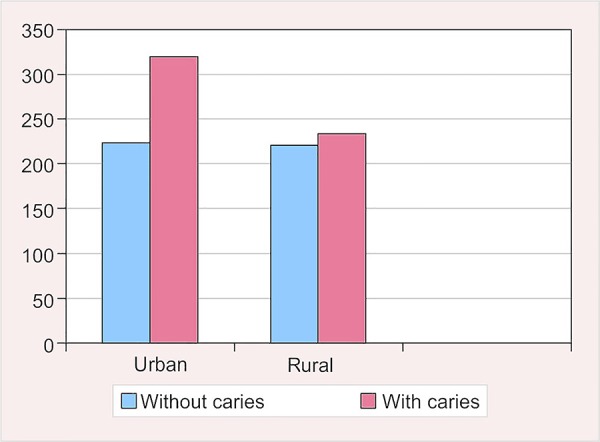
Prevalence of caries according to location

The prevalence of dental caries was found to be 58.9% among the urban population, while it was 51.4% among the rural population. The mean dmft and DMFT scores for the urban population were found to be 1.91 ± 2.11 and 1.19 ± 1.34 respectively. For rural population, the mean dmft and DMFT were recorded as 1.62 ± 2.11 and 0.82 ± 1.23 respectively (Table 3 and Fig. 3).

**Table 2:** **Table 2:** Prevalence of dental caries according to sex

				*Children free of caries*				*Children with caries*				*dmft*				*DMFT*			
*Gender*		*No. of children examined*		*N*		*%*		*N*		*%*		*Mean*		*SD*		*Mean*		*SD*	
Male		501		204		40.7		297		59.3		1.92		2.09		1.21		1.35	
Female		499		241		48.3		258		51.7		1.63		2.13		0.85		1.23	

SD: Standard deviation; Caries prevalence: Chi-square test = 5.813, p = 0.016; dmft: t-test = 2.176, p = 0.030; DMFT: t-test = 4.414, p = 0.000

**Table 3:** **Table 3:** Prevalence of dental caries according to location

				*Children free of caries*				*Children with caries*				*dmft*				*DMFT*			
*Location*		*No. of children examined*		*N*		*%*		*N*		*%*		*Mean*		*SD*		*Mean*		*SD*	
Urban		545		224		41.1		321		58.9		1.91		2.11		1.19		1.34	
Rural		455		221		48.6		234		51.4		1.62		2.11		0.82		1.23	

SD: Standard deviation; Caries prevalence: Chi-square test = 5.603, p = 0.018; dmft: t-test = 2.149, p = 0.032; DMFT: t-test = 4.525, p = 0.000

The difference in prevalence of dental caries between the urban and rural population was found to be statistically significant (p = 0.018). Similarly, the difference in the dmft (p = 0.032) and DMFT (p = 0.000) was found to be statistically significant.

### Prevalence of Dental Caries According to Socioeconomic Level

Of the study children, 61.8% in the low-socioeconomic-level group had caries in comparison with the 49.1% in the high-socioeconomic-level group. This difference was statistically very highly significant (p = 0.000). The dmft scores of children belonging to the low-socioeconomic level (2.13 ± 2.27) were considerably higher than that in the high-socioeconomic-level group (1.42 ± 1.89). The difference in the dmft scores between the two groups was very highly significant (p = 0.000).

**Fig. 4:**
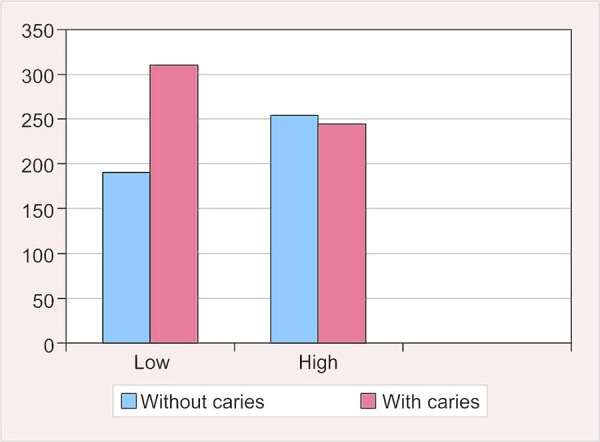
Prevalence of caries according to socioeconomic status

The mean DMFT of the low socioeconomic group children was 1.24 ± 1.34, whereas for the high socioeconomic group, it was 0.80 ± 1.22. The results indicated a very highly significant difference in between the two groups (p = 0.000) (Table 4 and Fig. 4).

**Table 4:** **Table 4:** Prevalence of dental caries according to socioeconomic (S-E) status

				*Children free of caries*				*Children with caries*				*dmft*				*DMFT*			
*S-E status*		*No. of children examined*		*N*		*%*		*N*		*%*		*Mean*		*SD*		*Mean*		*SD*	
Low		503		192		38.2		311		61.8		2.13		2.27		1.24		1.34	
High		497		253		50.9		244		49.1		1.42		1.89		0.80		1.22	

SD: Standard deviation; Caries prevalence: Chi-square test = 16.415, p = 0.000; dmft: t-test = 5.301, p = 0.000; DMFT: t-test = 5.418, p = 0.000

## DISCUSSION

Vaishali in the modern time is located in the state of Bihar, India. Even before the advent of Buddhism and Jainism, Vaishali was a vibrant republican state; in fact, it was the first republic in the world, similar to those later found in ancient Greece. In the Republic of Vaishali, Lord Mahaveer Jain was born. Buddha delivered his last sermon at Vaishali and announced his Nirvana here. Vaishali comprises mostly of rural population (total population is 21,46,065 with the rural share of 20,02,708)^4^ untouched by any preventive and educational dental health and expressed poor knowledge about oral hygiene awareness. Considering the poor lifestyle and unavailability of dental health services, the prevalence of dental diseases is expected to be high in this region. To deal with this problem successfully, it is essential to determine the extent and the severity of the problem. With these aims and objectives, the current study was conducted in Vaishali.

In the present study, the caries prevalence in the 5- to 7-year age group was found to be 65.1%, whereas in 8-to 10-year age group, it was 56.7%. In 11- to 13-year age group, it was the least, i.e., 45.4% (Table 1 and Fig. 1). It can be observed from the results obtained that the prevalence of caries decreased as the age advanced. The findings of this study are in accordance with the results obtained by Vaish,^5^ who showed a similar trend with prevalence rates of 58.3% in 6- to 8-year age group, 47.7% in 9- to 11-year age group and 44.3% in 12- to 14-year age group.

This decline in the caries rate with age may be due to the fact that there is increase in awareness of oral hygiene with age. This is supported by the finding of Chu et al.^6^ who found that 44 and 22% of the 6- and 12-year-old children respectively, had never brushed in their study population showing improvement in oral hygiene practices with age.

The difference in the dmft/DMFT among the three age groups in the study was found to be very highly significant. The mean dmft score declined progressively as the age advanced, whereas the mean DMFT score increased with age (Table 1). The decrease in the dmft values might be attributed to the reduction in the number of primary teeth with age due to normal exfoliation. The increase in DMFT may be coinciding with the eruption of permanent teeth. Rao et al.^1^ also reported a similar trend with the deft declining (4.53 ± 4.15 in 5- to 6-year age group to 1.81 ± 1.88 in 11- to 12-year age group) and DMFT increasing with age.

### Prevalence of Dental Caries According to Sex

In the present study, the difference in the prevalence of dental caries among the sexes has been found to be statistically significant. The prevalence rate found was 59.3% in males, whereas it was 51.7% in females (Table 2 and Fig. 2).

Various other authors including Vacher,^7^ Aukland and Bjelkaroey,^8^ and Joshi et al.^9^ observed a similar trend with higher caries experience in boys than in girls. Similarly, Kosovic and Nilsson-Andersson^10^ found more number of girls to be caries-free than the boys at 5 and 12 years in a survey conducted by them in Gambia. On average, in the group of 5-year-old boys, 3% were caries-free compared with 12% of girls. Corresponding values for 12-year-old boys and girls were 38 and 47% respectively. Another study done by Gangwar et al.^11^ reported the prevalence of dental caries to be 39.5% in males, whereas it was 36.5% in female children.

On the contrary, girls were found to have higher caries prevalence by Mishra and Shee^12^ and Saimbi et al.^13^ Shetty and Tandon^14^ and Addo-Yobo et al.^15^ found no difference in caries prevalence between boys and girls. This wide variation observed among different studies may be attributed to the different age groups and geographic locations studied in the surveys.

### Prevalence of Dental Caries According to Location

In the present study, the prevalence of dental caries in the urban population was found to be 58.9%, whereas in the rural population, it was 51.4% (Table 3 and Fig. 3). These findings were similar to the results obtained from the survey conducted by Subrata and Subrata.^16^ They observed 69.0% caries prevalence at 6 years of age in urban population, whereas it was 54.3% for the rural population. Higher caries prevalence among the urban population may be due to their easier accessibility to food items rich in refined sugar and reduced intake of coarse food in their diet. The result of our survey was also supported by the results observed by Addo-Yobo et al.^15^ who conducted a survey in Ghana and found the prevalence of dental caries to be 32% in the urban population and 12% in the rural population. A similar trend of caries prevalence was also reported by Chikte et al.^17^ and Li et al.^18^

### Prevalence of Dental Caries According to Socioeconomic Status

Determination of social class is complicated, especially in developing countries like India, due to nonexistence of clearly defined criteria. In our study, the study population was divided into two groups based on the socioeconomic level. Children belonging to the low socioeconomic group were those studying in the government schools and the high socioeconomic group comprised of children studying in public schools. The basis of division was similar to the one used by Kuriakose and Joseph^19^ who had divided their study population into three groups: Low, middle, and high. The low-income group children were from Anganwadis, the middle-income group children were from the state-run schools, and the high-income group children were from private nursery schools.

The present study has shown a significantly higher prevalence of dental caries among low socioeconomic group as compared with high socioeconomic group. The prevalence value was 61.8% for the low socioeconomic status group (mean dmft 2.13 ± 2.27; mean DMFT 1.24 ± 1.34), whereas it was 49.1% for the high socioeconomic group (mean dmft 1.42 ± 1.89; mean DMFT 0.80 ± 1.22) (Table 4 and Fig. 4). The difference was found to be highly significant statistically. These findings were in accordance with the observations of Singh et al.^20^ and Gillcrist et al.^21^ Powell et al.^22^ reported that the children from high-socioeconomic-level schools exhibited lower dmft and DMFT at all ages. At age 6 years, the proportion of children with caries-free primary dentitions for high-socioeconomic-level schools was 70%, for middle-socioeconomic-level schools, it was 52%, and for low-socioeconomic-level schools, it was 23%. The proportion of children with caries-free permanent dentition at 12 years for high, middle and low-socioeconomic-level groups was 35, 34, and 15% respectively.

Chandra and Chawla,^23^ on the contrary, observed higher caries prevalence in children belonging to the high socioeconomic status. Ghandour^24^ classified children into three socioeconomic groups: Low, middle, and high, but did not find any statistically significant difference between the caries prevalence of these groups.

The grouping of subjects according to the socioeconomic status encompasses the influence of income, education, and social environment.
